# Anatomical Locations of Keratinocyte Carcinomas Among United States Veterans Affairs Healthcare System Patients in Minnesota: A Cross‐Sectional Study

**DOI:** 10.1111/exd.70196

**Published:** 2025-12-19

**Authors:** Mikhail Usovich, John Meisenheimer, Madeeha Rizvi, Katelyn J. Rypka, Liang Liu, Erin M. Warshaw, Amy Gravely, Julie Lynch, Anders D. Westanmo, Noah Goldfarb

**Affiliations:** ^1^ Department of Dermatology Minneapolis VA Medical Health Care System Minneapolis Minnesota USA; ^2^ Department of Dermatology University of Minnesota Minneapolis Minnesota USA; ^3^ The Hormel Institute, University of Minnesota Minneapolis Minnesota USA; ^4^ From the Contact Dermatitis Clinic Park Nicollet Health System Minneapolis Minnesota USA; ^5^ Department of Research Minneapolis VA Medical Center Minneapolis Minnesota USA; ^6^ VA Informatics and Computing Infrastructure (VINCI), VA Salt Lake City Health Care System Salt Lake City Utah USA; ^7^ Division of Epidemiology, Department of Internal Medicine University of Utah School of Medicine Salt Lake City Utah USA; ^8^ Department of Pharmacy Benefits Services Minneapolis VA Health Care System Minneapolis Minnesota USA; ^9^ Department of Internal Medicine Minneapolis VA Health Care System Minneapolis Minnesota USA

**Keywords:** anatomical distribution, basal cell carcinoma, invasive squamous cell carcinoma, keratinocyte carcinoma, non‐melanoma skin cancer, skin cancer, squamous cell carcinoma in situ, veterans affairs healthcare system

## Abstract

Previous studies have demonstrated a higher prevalence of keratinocyte carcinomas (KC) on sun‐exposed areas, particularly for squamous cell carcinoma (SCC) compared to basal cell carcinoma (BCC). Few studies in the United States (US) have compared the prevalence of BCC to SCC by anatomical location. The aim of this study was to determine the KC prevalence in a Midwestern US population standardised by relative tumour density (RTD) per anatomical area, which has not been previously reported in the US. Data was collected from Veterans Affairs' patients with biopsy‐proven KCs from October 1999 to September 2020. KCs were divided into BCCs and SCCs. RTD was calculated by standardising frequency of occurrence in a location by body surface area. The proportion of BCCs to SCCs for each location was also analysed. A total of 31 663 KCs (17 776 BCCs and 13 887 SCCs) were identified in 10 933 patients, with the majority on the head and neck. BCCs were more likely overall on the trunk and upper arms than SCCs. SCCs were more common on the hands, forearms, genitals/perineum/perianal area, and ears. Both BCC and SCC occurred more frequently over sun‐exposed areas when controlling for body surface area of the location. Higher proportions of BCCs were found on sun‐protected areas when compared to SCCs. The relative preponderance of SCCs on the genitals/perineum/perianal area may be due to human papillomavirus.

**Abbreviations**
BCC: basal cell carcinoma
HPV: human papillomavirus
KC: keratinocyte carcinoma
RTD: relative tumour density
SCC: squamous cell carcinoma
US: United States
UV: ultraviolet
VAHCS: veterans affairs healthcare system

## 1 Introduction

Keratinocyte carcinoma (KC), which includes basal cell carcinoma (BCC) and squamous cell carcinoma (SCC), is the most common type of cancer worldwide. Each year in the United States (US), approximately 2 700 000 people are diagnosed with BCC, and 700 000 people are diagnosed with SCC [1, 2]. Ultraviolet (UV) light exposure from the sun is widely considered a major risk factor for the development of KC, although anatomical distribution differs between BCC and SCC [3, 4, 5, 6]. SCCs typically arise on habitually sun‐exposed areas of the body such as the scalp, forearms, and dorsal hands [3]. Conversely, a considerable percentage of BCCs are located on sun‐protected areas [4]. A literature search found few studies comparing BCC and SCC ratios in the United States (US). Only one US study calculated relative tumour densities (RTDs), which accounts for differences in surface area of anatomic regions, and only four anatomical locations had RTDs calculated [7, 8, 9, 10, 11]. In addition, no studies evaluated genitals/perineum/perianal areas independently from other sites. This study aimed to evaluate differences in anatomical distribution comparing BCCs with SCCs in a large, single‐site sample population of Veterans in the US Veterans Affairs Healthcare System (VAHCS) in Minneapolis, Minnesota.

## 2 Methods

This study was approved by the Minneapolis VA Institutional Review Board (IRB #1594484–6). Data was obtained from the Corporate Data Warehouse, which contains longitudinal electronic health record (EHR) data for Veterans, and compiled by the VA Informatics and Computing Infrastructure (VINCI). Information was collected on VAHCS patient demographic and biopsy data between October 1, 1999, and September 1, 2020, for individuals who were 18 years of age or older and who had at least one biopsy‐proven diagnosis of a KC.

KCs were divided into BCCs and SCCs. SCCs included both invasive squamous cell carcinoma and squamous cell carcinoma in situ. Frequencies of BCCs and SCCs were compared across different anatomical locations, including the head/neck, trunk, upper limbs, lower limbs, and genitals/perineum/perianal regions. These locations were further subdivided. Additionally, we calculated the RTD for these anatomical sites. RTD was defined as the ratio of tumours in an anatomical site to the total tumours of that type, divided by the skin surface area at that location. Skin surface area for each anatomical location was approximated and applied to all patients equally (Table 2). The estimations for skin surface area in each location were derived from the article by Subramaniam et al., with 1% skin surface area subtracted from the chest and/or abdomen and 1% skin surface area subtracted from the back and/or buttocks to account for the 2% skin surface area of the groin, perineum, and perianal area [3]. Ratios of BCCs to SCCs were also calculated for each anatomical site. RTD and ratios for BCCs to SCCs were analysed with a statistical significance of *α* < 0.05. Bootstrapping (2000 resamples with a size of 31 663 randomly selected samples) was utilised to produce 95% confidence intervals for these ratios. Statistical analysis was conducted using SAS Software Version 9.4 (Cary, North Carolina, US).

## 3 Results

There were 31 663 KCs identified in 10 933 predominantly male (98.0%) and White (98.7%) Veterans Affairs patients with a mean age of 72.7 years (Table 1). Of these KCs, 17 776 (56.1%) were BCCs, and 13 887 (43.9%) were SCCs.

**TABLE 1:** BCC and SCC demographics by person.

Variable	Total	BCC	SCC
	*N* = 10 933 (%)	*N* = 7914 (%)	*N* = 5843 (%)
Age			
Mean	72.7	72.2	74.1
Standard deviation	10.3	10.5	9.8
Sex			
Female	215 (2.0)	152 (1.9)	91 (1.6)
Male	10 718 (98.0)	7762 (98.1)	5752 (98.4)
Race			
White	9190 (98.7)	6650 (98.8)	4960 (98.7)
Black	9 (0.1)	2 (< 0.1)	7 (0.1)
Pacific Islander/Native Hawaiian	67 (0.7)	48 (0.7)	39 (0.8)
American Indian/Alaskan Native	42 (0.5)	31 (0.5)	21 (0.4)
Number missing	1625	1183	816
Ethnicity			
Not Hispanic	9717 (99.7)	7029 (99.7)	5261 (99.7)
Hispanic	27 (0.3)	18 (0.3)	16 (0.3)
Number missing	1189	867	566

Abbreviations: BCC, basal cell carcinoma; SCC, squamous cell carcinoma. The number of people with BCC and SCC do not add to the number in the total column due to some people having both SCC and BCC.

The most common locations for SCCs were the face (35.0%), ears (12.0%), scalp (9.9%), hands (9.2%), and forearms (9.1%). Conversely, BCCs were most commonly located on the face (47.9%), back/buttocks (12.9%), and upper arms (9.7%). Regarding RTD, the highest densities of BCCs were observed on the face, ears, neck, and upper arms, whereas SCCs had the highest RTD on the ears, face, and hands (Table 2).

**TABLE 2:** Proportions, relative tumour densities, and ratios.

Body site (body surface area %)	BCC *N* = 17 776 (%)	RTD (95% CI)^a^	Total SCC *N* = 13 887 (%)	RTD (95% CI)^a^	BCC:SCC ratio (95% CI)
Head and/or neck (9.0)	11 431 (64.3)	7.15 (7.07, 7.22)	8637 (62.2)	6.91 (6.82, 7.00)	1.03 (1.01, 1.06)
Scalp (3.7)	586 (3.3)	0.89 (0.62, 0.96)	1375 (9.9)	2.68 (2.54, 2.81)	0.33 (0.30, 0.37)
Ears (0.5)	1116 (6.3)	12.56 (11.84, 13.27)	1666 (12.0)	23.99 (22.91, 25.07)	0.52 (0.49, 0.56)
Face (2.4)	8512 (47.9)	19.95 (19.65, 20.26)	4858 (35.0)	14.58 (14.26, 14.91)	1.37 (1.32, 1.42)
Neck (2.4)	1099 (6.2)	2.58 (2.43, 2.72)	627 (4.5)	1.88 (1.74, 2.03)	1.37 (1.25, 1.51)
Head NOS (NA)	118 (0.66)	—	111 (0.8)	—	0.83 (0.64, 1.09)
Trunk (30.0)	3507 (19.7)	0.66 (0.64, 0.68)	1255 (9.0)	0.30 (0.29, 0.32)	2.18 (2.05, 2.33)
Chest and/or abdomen (12.0)	1208 (6.8)	0.57 (0.54, 0.60)	675 (4.9)	0.41 (0.38, 0.43)	1.40 (1.28, 1.54)
Back and/or buttocks (18.0)	2299 (12.9)	0.72 (0.69, 0.75)	580 (4.2)	0.23 (0.21, 0.25)	3.10 (2.83, 3.39)
Upper limbs (19.0)	2329 (13.1)	0.69 (0.66, 0.72)	3486 (25.1)	1.32 (1.28, 1.36)	0.52 (0.50, 0.55)
Upper arm (8.0)	1731 (9.7)	1.22 (1.16, 1.27)	952 (6.9)	0.86 (0.80, 0.91)	1.42 (1.31, 1.54)
Forearm (6.0)	542 (3.1)	0.51 (0.47, 0.55)	1264 (9.1)	1.52 (1.44, 1.60)	0.34 (0.30, 0.37)
Hand^b^ (5.0)	56 (0.3)	0.06 (0.05, 0.08)	1270 (9.2)	1.83 (1.73, 1.92)	0.04 (0.03, 0.04)
Lower limbs (40.0)	500 (2.8)	0.07 (0.06, 0.08)	457 (3.3)	0.08 (0.07, 0.09)	0.86 (0.75, 0.98)
Thigh (19.0)	111 (0.6)	0.03 (0.03, 0.04)	101 (0.7)	0.04 (0.03, 0.05)	0.86 (0.65, 1.12)
Lower leg (14.0)	378 (2.1)	0.15 (0.14, 0.17)	338 (2.4)	0.17 (0.16, 0.19)	0.87 (0.76, 1.02)
Foot^c^ (7.0)	11 (0.1)	0.01 (0.00, 0.01)	18 (0.1)	0.02 (0.01, 0.03)	0.48 (0.20, 1.02)
Genitals/perineum/perianal (2.0)	9 (0.1)	0.03 (0.01, 0.04)	52 (0.4)	0.19 (0.14, 0.24)	0.14 (0.06, 0.24)

*Note:* While the majority of head and/or neck biopsies specified specific locations, a few were categorised as head only. These lesions were categorised as head not otherwise specified (NOS). As no estimated body surface area was associated with this subcategory, relative tumour densities were not calculated for tumours in this category. Genitals/perineum/perianal is not broken intosubcategories. Percentages were rounded to the first decimal place, and therefore may not add up to 100%.

Abbreviations: BCC, basal cell carcinoma; NOS, not otherwise specified; RTD, relative tumour density; SCC, squamous cell carcinoma.

^a^ Calculated as the ratio of the proportion of tumours at a specific anatomical site to the proportion of skin surface area at that site.

^b^ Includes back of hand, palmar skin, and fingernail.

^c^ Includes dorsum of foot, toes, plantar skin, and toenail.

The BCC‐to‐SCC ratios varied significantly by anatomical location. BCCs were more prevalent on the chest/abdomen (1.40; 95% CI 1.28–1.54), back/buttocks (3.10; 95% CI 2.83–3.39), and upper arms (1.42; 95% CI 1.31–1.54). In contrast, SCCs predominated on the scalp (0.33; 95% CI 0.30–0.37), ears (0.52; 95% CI 0.49–0.56), forearms (0.34; 95% CI 0.30–0.37), hands (0.04; 95% CI 0.03–0.04), and genitals/perineum/perianal areas (0.14; 95% CI 0.06–0.24).

## 4 Discussion

As previously noted by Subramaniam et al., SCC distribution seems more heavily influenced by UV light exposure than BCC distribution, supported by the lower ratio of BCC to SCC in sun‐exposed areas [3]. For the study herein, the greatest incongruence between SCC and BCC locations was on the hands, with a nearly 29‐fold greater incidence of SCCs over BCCs. This discrepancy supports a greater influence of chronic UV exposure on the development of SCC when compared to BCC [3]. It is important to note that while the ratio of BCC to SCC was lower in sun‐exposed areas, both BCC and SCC were more common in sun‐exposed than sun‐protected areas.

It has been suggested that the discrepancy in the location of SCCs and BCCs is due to a greater contribution of severe sunburns in the pathogenesis of BCC in sun‐protected areas, but there are contradictory findings on this topic, with some studies suggesting intermittent sun exposure is an equal risk factor for both SCCs and BCCs [12, 13]. An alternative explanation is that chronic UV exposure results in persistent, localised immunosuppression, increasing the risk of SCC in comparison to BCC in sun‐exposed areas, given the risk of SCC increases proportionally with the degree of immunosuppression in solid organ transplant patients [14, 15].

Interestingly, the genitals/perineal/perianal areas had a higher proportion of SCCs. Given the minimal amount of UV exposure in these areas, the development of skin cancer may be influenced by other factors, like human papillomavirus [16, 17]. Future studies evaluating the role of HPV infection in genital SCC would be beneficial.

This study's use of RTD provides a more precise understanding of KC distribution by accounting for variations in body surface area, offering a standardised metric for comparing tumour burden across different anatomical locations. The large sample size and comprehensive dataset, derived from over two decades of VAHCS records, strengthen the validity of our findings. However, the retrospective design introduces potential biases, such as differential biopsy practices based on anatomical location and perceived cancer risk. Lesions on the head and neck, for example, may be biopsied more frequently due to the perception of being a high‐risk location. Moreover, the study's predominantly male, White population limits the generalizability of the findings to other demographic groups.

## 5 Conclusion

This study provides a detailed analysis of KC distribution across various anatomical regions, utilising RTD to offer a standardised comparison of BCC and SCC occurrences. This study also highlights the potential role of non‐UV factors, such as HPV, in SCC development in the genitals/perineum/perianal areas. Clinicians should be mindful of these distinctions in tumour distribution when assessing patients for suspicious lesions and performing biopsies. Future research should focus on expanding these findings to more diverse populations, exploring the molecular mechanisms underlying BCC and SCC pathogenesis, and identifying potential preventative strategies for both UV‐related and non‐UV‐related KCs.

## Author Contributions

Mikhail Usovich contributed to the study design, data collection, and manuscript writing. John Meisenheimer contributed to the manuscript's writing. Madeeha Rizvi contributed to the manuscript's writing. Katelyn J. Rypka contributed to the data collection. Liang Liu contributed to the study design and manuscript writing. Erin M. Warshaw contributed to the study design and manuscript writing. Amy Gravely contributed to the data analysis. Julie Lynch contributed essential data collection resources. Anders D. Westanmo contributed to the study design and data collection. Noah Goldfarb contributed to the study design and manuscript writing. M.U., J.M., M.R., K.J.R., L.L. E.M.W., A.G., J.L., A.D.W., and N.G. have read and approved the final manuscript.

## Funding

The authors have nothing to report.

## Disclosure

The content of this publication does not represent the views of the Department of Veterans Affairs or the United States Government.

## Ethics Statement

This study was approved by IRB #1594484‐6.

## Conflicts of Interest

Dr. Goldfarb has participated in clinical trials with Abbvie, Pfizer, Chemocentrix, and DeepX Health. He has also served on advisory boards and consulted for Novartis, UCB, and Boehringer Ingelheim. Dr. Lynch reports grants from Alnylam Pharmaceuticals Inc., Astellas Pharma Inc., AstraZeneca Pharmaceuticals LP, Biodesix Inc., Celgene Corporation, Cerner Enviza, GSK PLC, IQVIA Inc., Janssen Pharmaceuticals Inc., Kantar Health, Myriad Genetic Laboratories Inc., Novartis International AG, Parexel International Corporation through the University of Utah or Western Institute for Veteran Research outside the submitted work. Mr. Usovich has no conflicts to report. Dr. Westanmo has no conflicts to report. Dr. Meisenheimer has no conflicts to report. Ms. Rizvi has no conflicts to report. Dr. Rypka has no conflicts to report. Dr. Warshaw has no conflicts to report. Mrs. Gravely has no conflicts to report. Dr. Liu has no conflicts to report.

## Data Availability

The data that support the findings of this study are available from the corresponding author upon reasonable request.
